# Reinforcement and MAO-A inhibition in heated tobacco products: flavor and brand variations

**DOI:** 10.3389/fpsyt.2025.1515519

**Published:** 2025-02-18

**Authors:** Xiangyu Li, Zheng Ding, Xingyi Jiang, Hongjuan Wang, Yanbo Luo, Huan Chen, Yongqiang Pang, Hongwei Hou, Qingyuan Hu

**Affiliations:** ^1^ China National Tobacco Quality Supervision &Test Center, Zhengzhou, Henan, China; ^2^ Key Laboratory of Tobacco Biological Effects, Zhengzhou, Henan, China; ^3^ Key Laboratory of Precision Nutrition and Food Quality, Department of Nutrition and Health, China Agricultural University, Beijing, China

**Keywords:** heated tobacco products (HTPs), self-administration, MAO-A inhibition, MAO inhibitors, nicotine reinforcement

## Abstract

**Objective:**

This study investigates the reinforcing effects and monoamine oxidase-A (MAO-A) inhibitory properties of heated tobacco products (HTPs), comparing them to nicotine alone. It also examines brand-specific differences in MAO-A inhibition to provide a deeper understanding of the role of non-nicotine constituents in HTP use.

**Methods:**

A rat self-administration model was used to evaluate the reinforcement patterns of HTP-T (tobacco flavor), HTP-M (menthol flavor), and nicotine under fixed-ratio schedules. *In vitro* assays were performed to measure the MAO-A inhibitory effects of nicotine, HTP-T, and HTP-M. Additionally, chemical composition analyses of HTP-T and HTP-M aerosols were conducted and compared to identify potential MAO inhibitors. Finally, *in vitro* assessments of MAO-A inhibition were performed across various HTP brands to determine whether significant differences in MAO-A inhibition exist among different HTP products.

**Results:**

HTP-T showed self-administration patterns comparable to nicotine, while HTP-M demonstrated significantly higher reinforcement. *In vitro* analyses revealed that both HTP-T and HTP-M exhibited MAO-A inhibition at high nicotine-equivalent concentrations (>10^−2^–10^−1^ mM), with HTP-M showing stronger inhibition. In contrast, Nicotine alone showed no MAO-A inhibition. Brand-specific differences in MAO-A inhibitory effects were also observed, potentially driven by variations in aerosol composition.

**Conclusions:**

HTP-M's enhanced reinforcement could be attributed to its higher MAO-A inhibition and menthol's synergistic effects on nicotine. Brand-specific variations in MAO inhibition highlight the impact of non-nicotine constituents on HTP use. While this study provides valuable insights into HTPs, its reliance on animal models and *in vitro* assays highlights the necessity for human studies conducted under real-world conditions.

## 1 Introduction

Heated Tobacco Products (HTPs), which are increasingly popular as alternatives to conventional cigarettes, has been actively promoted by industry manufacturers (1). Given the potential health risks associated with HTPs, exploring their use patterns is critical for public health, especially since they can deliver nicotine, a substance that plays a central role in tobacco addiction and use persistence (2). One WHO report points out that switch studies from conventional cigarettes to HTPs showed increases in both HTP usage and nicotine consumption over time, highlighting the need for a thorough evaluation of HTP use (3).

Research related to HTP usage has mainly focused on nicotine emissions, exposure biomarkers, and pharmacokinetics (4, 5). In comparative analyses of nicotine exposure from HTPs and conventional cigarettes, serum cotinine levels and nicotine equivalents in 24-hour urine samples are commonly used as nicotine biomarkers (6). The study by Biondi-Zoccai et al. (7) reported a plasma cotinine increase of 30.6 ng/mL for iQOS, comparable to 31.1 ng/mL for conventional cigarettes. Similarly, another study found that urinary nicotine equivalents in Glo users ranged from 59% to 74% of those in conventional cigarette users (8). While BAT and PMI studies suggested similar pharmacokinetics between HTPs and conventional cigarettes (8, 9), other research showed HTPs have a shorter time to maximum plasma nicotine concentration (T_max_) and lower nicotine delivery, potentially increasing their addictive potential (10, 11). However, these pharmacokinetic findings remain inconclusive, highlighting the need for further independent studies.

HTP aerosol is generated through medium-temperature (<350~400°C) heating of tobacco substrates. This process contrasts with the high-temperature, combustion-driven mechanisms observed in conventional cigarettes (4). This distinction in aerosol generation leads to a chemical profile of HTPs that is fundamentally different from that of conventional cigarettes. Beyond nicotine, other chemical constituents in HTP aerosols may also influence their usage, highlighting the need for a broader evaluation of these factors. For instance, monoamine oxidase-A (MAO-A) inhibitors, which are present in cigarette smoke, are known to enhance the rewarding effects of nicotine by inhibiting MAO-A activity in the brain and subsequently increasing dopamine release (12). Previous studies have demonstrated that the inhibition of MAO-A, rather than MAO-B, enhances nicotine reinforcement in rats (13, 14).

Our hypothesis sought to determine whether MAO-A inhibition plays a role in HTP use and whether HTP products exhibit varying degrees of MAO-A inhibition compared to nicotine alone. However, a tobacco industry study reported no MAO inhibition by HTP aerosols *in vitro* (15). Considering non-nicotine components of tobacco emissions, such as MAO inhibitors, would provide a deeper understanding of HTP's use. To our knowledge, a significant gap exists in the comprehensive study of HTPs, particularly from the perspective of non-nicotine constituents (16). The deficiency is even more pronounced when it comes to exploring the brand-specific differences that may exist within this domain. A broader and more detailed investigation into MAO inhibition would help understand the complexities of HTP use.

To address this research gap, we conducted an animal self-administration study to compare the reinforcing effects of tobacco-flavored (HTP-T) and menthol-flavored (HTP-M) heated tobacco products (HTPs), alongside an *in vitro* comparison of their monoamine oxidase (MAO) inhibitory effects. In addition, we assessed MAO inhibition across various HTP brands to investigate potential brand-specific variations. Tobacco and menthol flavors were selected as focus of this study because data from Japan, the largest market for HTPs, indicate that these two flavors dominate consumer preferences and are the most widely consumed (17). The intravenous self-administration model is widely recognized as an effective and reliable tool for assessing the reinforcing effects of nicotine and has also been successfully used to evaluate the impact of MAO inhibition on nicotine reinforcement (14). Our preliminary findings suggest that menthol-flavored HTPs exhibit stronger reinforcement effects than tobacco-flavored HTPs and reveal substantial brand-specific variations in MAO inhibition.

## 2 Methodology

### 2.1 Material

Nicotine bi-L-(+)-tartrate dihydrate was procured from TCI (catalog number N0080). The MAO-Glo assay system was acquired from Promega (catalog number V1402). The recombinant human MAO-A, expressed in baculovirus infected BTI insect cells used in the study was purchased from Sigma-Aldrich (No. M7136). Various brands of heated tobacco products (HTPs) were purchased from international markets. The mainstream aerosols of all HTPs were produced using identical brand-specific heating devices. The main constituents (ACM, nicotine and menthol contents) of the mainstream aerosols for samples 1-9 are provided in **Supplementary Table S1** of the supporting information. For the self-administration trials, two flavors of HTPs—tobacco and menthol—were used, designated as HTP-T and HTP-M respectively. Both flavors were sourced from the same manufacturer and heated using an identical heating device to maintain uniformity.

### 2.2 Methods

#### 2.2.1 Animals

In the animal experiment, the inclusion and exclusion criteria were strictly adhered to, with no animals or data points excluded. The animals were randomly assigned to groups before the experiment. Male Sprague Dawley rats, aged 7 weeks with body weights ranging from 200–250 grams, were obtained from Beijing Vital River Laboratory Animal Technology Co., Ltd. Upon their arrival at our laboratory, the rats were accommodated with environmental controls for temperature, humidity, and atmospheric pressure. The light cycle was set from 8:00 p.m. to 8:00 a.m., aligning with their nocturnal activity period and our testing schedule. A one-week acclimation phase was allotted prior to initiating experimental procedures to allow the rats adjusting to the new surroundings. Each rat was individually housed and equipped with steel lids, primarily for food storage, and filter tops. They were granted unrestricted access to water and standard laboratory chow to ensure their well-being. With the initiation of the experimental phase, a controlled diet was implemented, providing approximately 10 grams (equivalent to 2-3 pellets) of food daily to maintain consistent body weight throughout the study period. All experimental protocols adhered strictly to the National Institutes of Health Guide for the Care and Use of Laboratory Animals. Blinding was maintained throughout the allocation, conduct, outcome assessment, and data analysis stages to minimize biases. All outcome measures were clearly defined, with the primary outcome measure specified for hypothesis-testing studies.

#### 2.2.2 MAO-A assay

This methodological approach was designed to elucidate the inhibitory effects within the MAO reactions under study. The detection of MAO-A activity was performed using the MAO-Glo™ Assay Systems (Promega, catalog number V1401). This bioluminescent two-step assay was executed using Nunc white 96-well, flat-bottom assay plates from Corning (catalog number YZ-3358). The assay involved incubating recombinant MAO-A enzyme (Sigma, catalog number M7316, 0.4 U/well of microsomal protein) with a beetle luciferin derivative—specifically, (4S)-4,5-dihydro-2-(6-hydroxybenzothiazolyl)-4-thiazolecarboxylic acid—alongside either the test substance or a control vehicle. Incubation was performed for 1 hour at room temperature in a 50-µL reaction mixture. To assess kinetic constants, the concentrations of the beetle luciferin substrate were varied. However, in subsequent experiments, the substrate concentration was standardized at its Michaelis constant (Km) of 20 µM. In the subsequent detection step, 50 µL of luciferin detection reagent was added to the MAO reaction mixture. Following an additional hour of incubation, the luminescent signal was measured using Tecan Spark Cyto Plate Reader (China Tecan laboratory equipment Co.,Ltd.).

#### 2.2.3 Generation of TPM/ACM extracts

Prior to use in the study, HTP sticks were conditioned in accordance with ISO Standard 3402. HTP aerosols were generated on a 6-port HTP smoking machine (Hefei Institutes of Physical Science, Chinese Academy of Sciences, catalog number SML600H, Hefei, China) in accordance with the Health Canada intense smoking protocol (puff volume: 55 mL; puff duration: 2s; puff frequency: 2 min^−1^; no blocking for tilter ventilation holes for HTPs, as per CORESTA Recommend Method No. 101) (18). HTP aerosol collected mass (ACM) was collected on Cambridge glass-fiber filters (44-mm diameter; Borgwaldt) and then extracted with 10 ml of dimethyl sulfoxide (DMSO). The solvent-free extracts of HTP ACM were extracted using the high-speed centrifugal method which has been proven to eliminating solvent influence and achieve high-dose exposure *in vitro* toxicology assessments. Comprehensive details can be found in Wang et al. (19). For the self-administration procedures, all HTP ACM extracts were diluted to a standardized nicotine concentration of 250 μg/mL and administered via intravenous injection. The key advantage of the intravenous self-administration model lies in its ability to simulate the pharmacokinetics of tobacco products, enabling controlled and precise nicotine administration.

In the *in vitro* MAO-A inhibition assays comparing nicotine, HTP-T, and HTP-M, two distinct methodologies were used to extract the main constituents from mainstream aerosols: traditional solvent extraction (SE) and physical centrifugal extraction (CE). These methods were chosen to enable a comprehensive comparison of their inhibitory effects on MAO-A. All extracted samples were diluted in a gradient based on nicotine concentration, ranging from 10^-5^ mM to 10^0^ mM nicotine/mL, to assess their MAO-A inhibitory effects. Since physical centrifugal extraction demonstrated stronger inhibitory effects, subsequent *in vitro* MAO-A inhibition experiments across different HTP brands were conducted exclusively using the centrifugal extraction method.

#### 2.2.4 Aerosol chemical composition analysis

The nicotine and menthol content in aerosol extracts was quantified using gas chromatography with flame ionization detection (GC-FID) on an Agilent 7890A system. An ACL-1 capillary column (30 m × 0.25 mm × 0.25 µm) was used, with helium as the carrier gas at a constant flow rate of 2.0 mL/min. The injection port and detector temperatures were set at 250°C, with a split injection mode (split ratio 40:1) and an injection volume of 1.0 µL. The oven temperature program was as follows: 100°C for 1 min, increased at 15°C/min to 220°C, and held for 6 min. This method ensured accurate and reproducible quantification of nicotine and menthol content in the aerosol samples.

The non-targeted chemical composition of HTP-T and HTP-M aerosol samples was analyzed using gas chromatography-quadrupole/orbitrap high-resolution mass spectrometry (GC-Q/Orbitrap-HRMS). A DB-5 capillary column (60 m × 0.25 mm × 0.25 μm, Agilent, USA) with helium as the carrier gas (1.0 mL/min) was used. The injection port was set at 280°C, with 1 μL of sample injected in split mode (15:1). The oven temperature program included the following steps: 50°C for 2 min, increased to 104°C at 6°C/min (held for 5 min), then to 164°C at 6°C/min (held for 4 min), and finally to 280°C at 6°C/min (held for 2 min).Mass spectrometric parameters included electron ionization (70 eV), an ion source and transfer line temperature of 280°C, full scan mode (33–495 Da), and a resolution of 60,000 FWHM (m/z 200) with lock mass calibration. Data were processed using Thermo Scientific™ TraceFinder and Compound Discoverer 3.3 software, involving peak retrieval, deconvolution, database search (NIST and Wiley libraries), and compound identification. This approach enabled a comprehensive comparison and differentiation of aerosol chemical constituents across samples.

#### 2.2.5 Self-administration procedures

Prior to the experiment, surgical instruments were sterilized using 75% ethanol and subsequently irradiated under ultraviolet light for 30 minutes. Rats were anesthetized with isoflurane and intramuscularly injected with Zoletil^TM^ 50 (Virbac). Hair from the right chest and back of each rat was removed using an electric shaver to prepare the surgical site. The pulsating jugular vein in the right chest of rat was located, and a pre-sterilized silicone tube was gently inserted. After securing the silicone tube, the other end of the tube was connected to the self-administration button (Instech, VABR1B/22) on the rat’s back. The self-administration button was protected by a special cover (Instech, VABRC-G) to prevent the magnet from rusting. To prevent infection and ensure patency, the silicone tubes were flushed daily with heparin sodium and penicillin solution. A recovery period extending to at least seven days was observed to allow the rats to heal adequately from the surgical procedure before they were introduced to the self-administration phase of the experiment. Notably, the self-administration sessions began without preliminary food training to avoid influencing the drug self-administration behavior of rats, as prior studies (20–22) have suggested that food training can increase self-administration in rodents.

The experiment employed fixed ratio (FR) schedules for self-administration, following the methodology outlined by Weiss et al. (23), where the FR value denotes the number of required responses for a single drug delivery. Specifically, the FR1, FR2, and FR3 schedules necessitated 1, 2, and 3 responses, respectively, for each drug infusion (24). This gradual increase in FR levels facilitated learning and adaptation to more demanding conditions. Activation of an infusion resulted in the chamber light being extinguished for 20 seconds, accompanied by a 1-second beep to report the delivery. During these 20 seconds, all additional nose pokes were recorded but considered non-contingent. The FR1 schedule was employed to facilitate the learning of self-administration behaviors in rats (25), while the FR2 and FR3 schedules explored the maintenance and reinforcement of drug intake (26, 27).

In the preliminary stage of the investigation, nicotine and saline were used as control to validate the robustness of the nicotine self-administration model. Fourteen rats were randomly divided into two groups (nicotine and saline), with seven rats in each group. For the comparative analysis of HTP-T (tobacco-flavored HTP) and HTP-M (menthol-flavored HTP), a cohort of 18 rats was used. These rats were randomly assigned to three groups: nicotine, HTP-T, and HTP-M, with six rats per group. Self-administration trials were conducted to evaluate and compare the reinforcing effects of the substances. In total, 32 rats were used across all phases of the self-administration experiments.

The experimental design of HTP dosage was carefully crafted to reflect dosages typical of human smokers. Previous pharmacokinetic studies of cigarettes have suggested that a nicotine dosage equivalent to human smoking behavior is around 30μg/kg/injection. Additionally, studies have shown comparable cotinine levels in the urine of HTP and conventional cigarettes users, underscoring the relevance of this dosage (28). In light of these findings, the self-administration dosages for all experimental groups were standardized to a nicotine concentration of 30 μg/kg per injection. The groups were categorized as follows: Saline (S), Nicotine (N, 30 μg/kg per injection), HTP-tobacco flavor (HTP-T, with nicotine dosage adjusted to 30 μg/kg per injection) and HTP-menthol flavor (HTP-M, with nicotine dosage adjusted to 30 μg/kg per injection). These dosages were consistently applied throughout the study, even as the required responses for drug delivery increased from fixed-ratio (FR) schedules FR1 through FR3 to examine drug intake behaviors and reinforcement.

### 2.3 Statistical analysis

Data analysis was predominantly executed through one-way Analysis of Variance (ANOVA) for repeated measures, with the addition of multiple comparison tests and t-tests applied as necessary. These analyses were conducted using GraphPad Prism version 8.0. In terms of data presentation, graphical illustrations display values as the mean ± Standard Error of the Mean (SEM), offering a concise visualization of the data's variability.

## 3 Results

### 3.1 Self-administration behaviors in animal models

It was noteworthy that rats in all groups successfully learned and performed self-administration via nose-poking in the FR1 schedule without preliminary food training, indicating a successful adaptation to the self-administration model. The data showed a clear propensity for active self-administration of nicotine in the N group (nicotine) compared to the S group (saline), particularly in the FR2 and FR3 phases, with significant statistical differences (P<0.05) (**Supplementary Figure S1**). The transition from FR1 to FR3 did not significantly alter the rate of nicotine-driven self-administration, indicating consistent maintenance of self-administration behaviors without significant enhancement over time. These results are consistent with existing literature, which demonstrates that nicotine significantly induces self-administration at doses related to human smoking due to its rewarding effects (29). These outcomes demonstrate the successful establishment of a nicotine self-administration model, with findings suggesting that nicotine reinforcing effect on self-administration is evident but somewhat limited.

**Figure 1:**
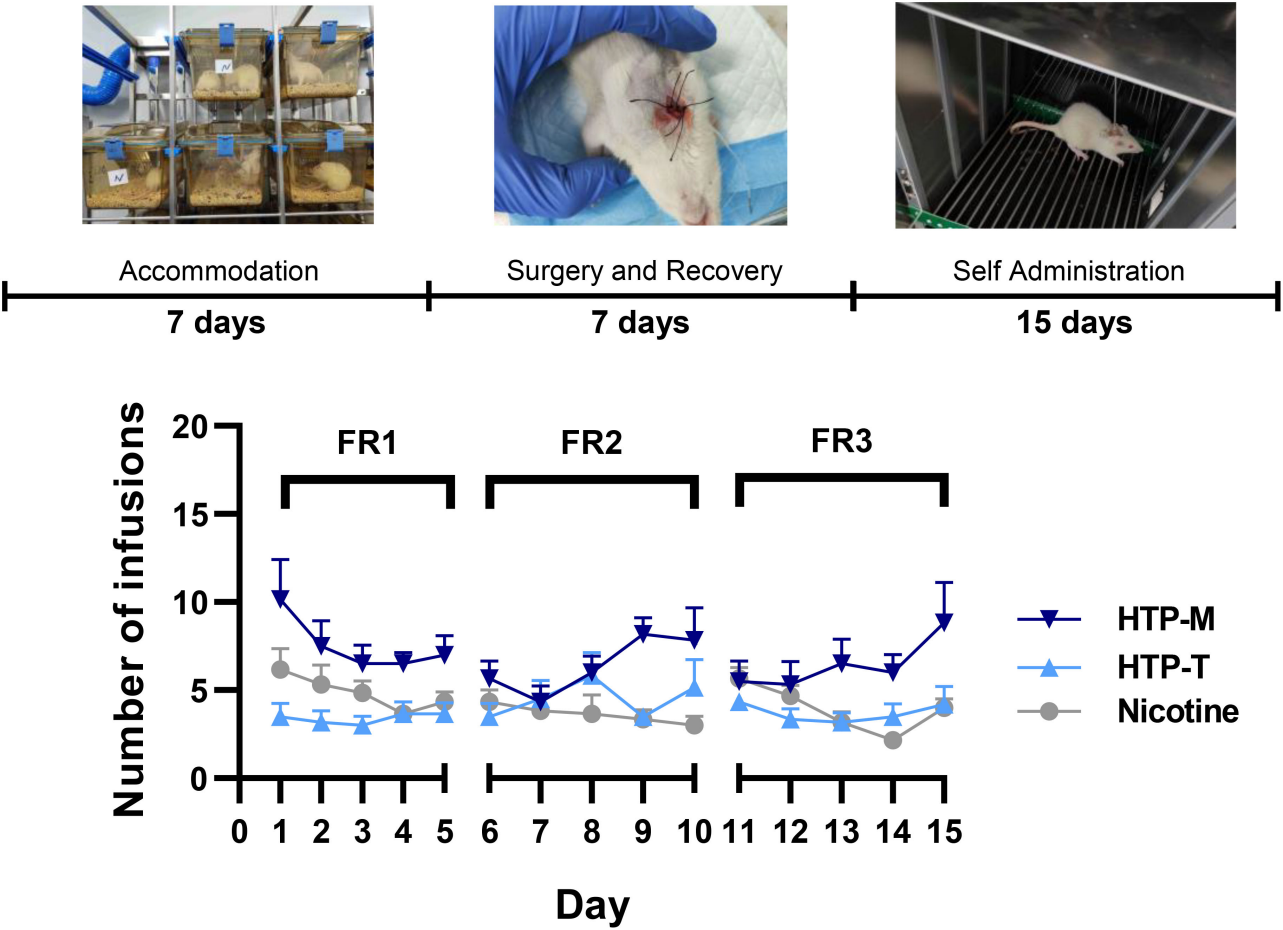
Self-administration rates across nicotine and Heated Tobacco Products (HTP-T and HTP-M) under progressive Fixed-Ratio schedules (FR1 From Day 1 to Day 5, FR2 from Day 6 to Day 10, FR3 from Day 11 to Day 15.

The above results demonstrate that the self-administration model is sufficient to maintain stable nicotine intake in mice for up to 27 days, but there are fluctuations starting from day 16. To address this, we optimized the timing of self-administration (**Figure 1**). Rats were placed in operant chambers for 1 hour with FR1, FR2, FR3 self-administration schedules on days 1–5, 6–10, and 11–15, respectively. The results from the self-administration experiments involving nicotine, HTP-T (tobacco flavor) and HTP-M (menthol flavor) comprehensively presented in **Figure 1**.

Nicotine and menthol testing results show that HTP-M aerosol contains 1.46 mg/stick of nicotine and 3.22 mg/stick of menthol, whereas HTP-T aerosol contains 1.65 mg/stick of nicotine and does not contain detectable levels of menthol. The self-administration level of the HTP-M group was significantly higher than that of the nicotine group, with a pronounced difference under the FR2 and FR3 schedule (p=0.0015). In contrast, the self-administration level of the HTP-T group under the FR2 and FR3 schedule was found to be slightly higher than that in the nicotine group, but the difference was not statistically significant (p>0.05), suggesting comparable levels of reinforcement.

Interestingly, HTP-M self-administration rate was not only higher than that of the nicotine group (P=0.0015), but also surpassed the HTP-T group (P=0.0027). The self-administration patterns were further compared among nicotine, HTP-T and HTP-M groups. For HTP-T group, the trend in self-administration was closely mirrored that observed in the nicotine group. In contrast, HTP-M group exhibited a pronounced increase in the FR2 & FR3 schedule, where both groups showed increased self-administration activity towards the final days (Days 9& 10 and Days 14 & 15).

### 3.2 Comparative analysis of monoamine oxidase-A inhibition by nicotine, HTP-T and HTP-M

**Figure 2:**
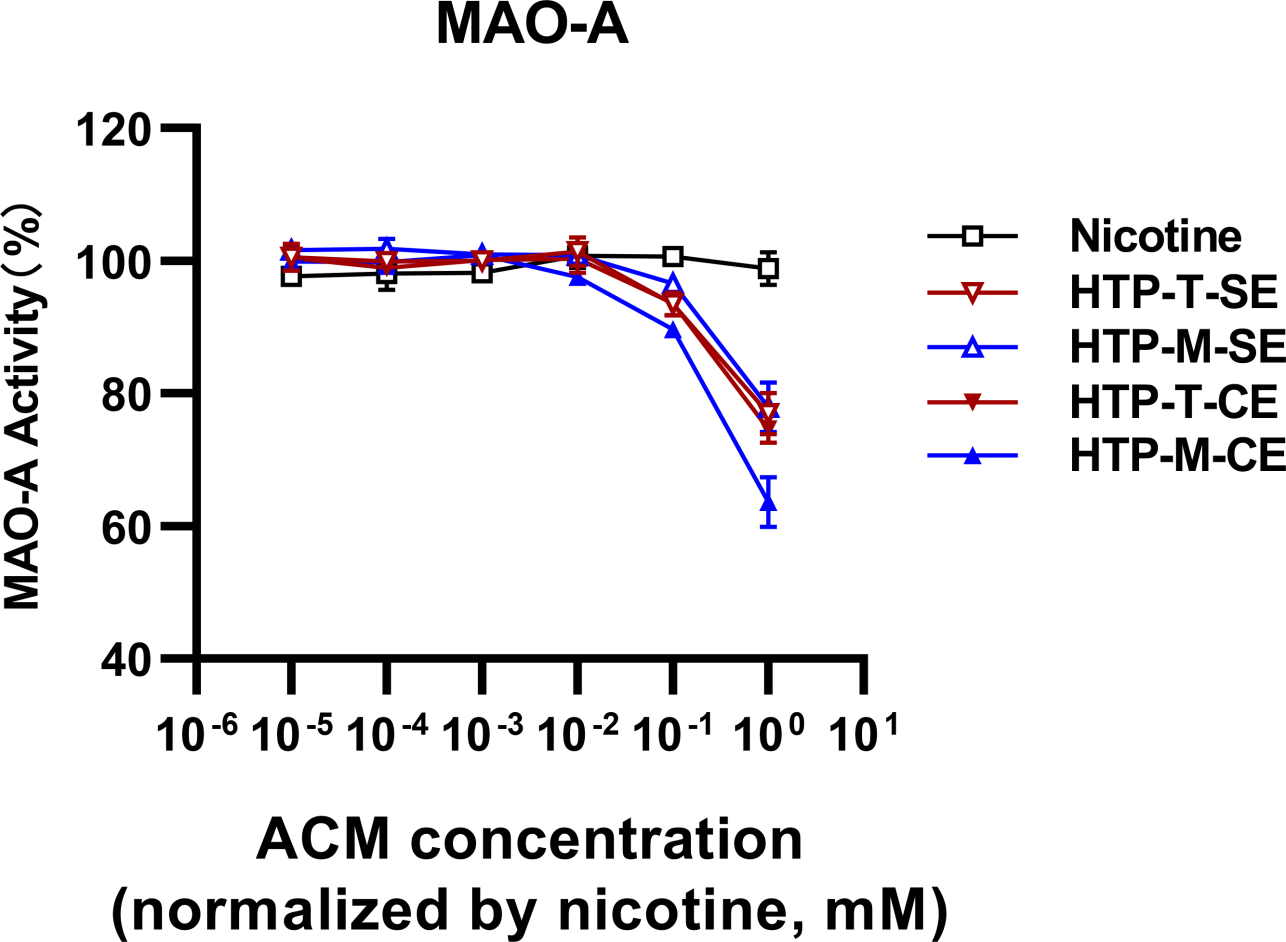
Analysis of MAO-A inhibition by heated tobacco products using two extraction techniques (SE, Solvent Extraction; CE, Centrifugal Extraction).

Our hypothesis aimed to determine whether MAO-A inhibition, played a role in the distinction between HTP-T and HTP-M. Furthermore, we aimed to assess whether HTP products could inhibit MAO-A to a different extent compared to nicotine alone (**Figure 2**). The samples extracted via centrifugal methods (labeled as CE in **Figure 2**) showed more pronounced inhibitory effects than those obtained through solvent extraction (labeled as SE in **Figure 2**), possibly indicating a more effective and less solvent-biased extraction process. As expected, nicotine alone exhibited no inhibitory effects on MAO-A at nicotine concentrations ranging from 10^-5^ to 10^0^ mM. However, both HTP samples started to demonstrate inhibitory effects within 10^-2^ to 10^-1^ mM range. Notably, HTP-M showed a greater degree of MAO-A inhibition across the entire 10^−2^; to 10^0^ mM nicotine concentration range compared to HTP-T.

**Figure 3:**
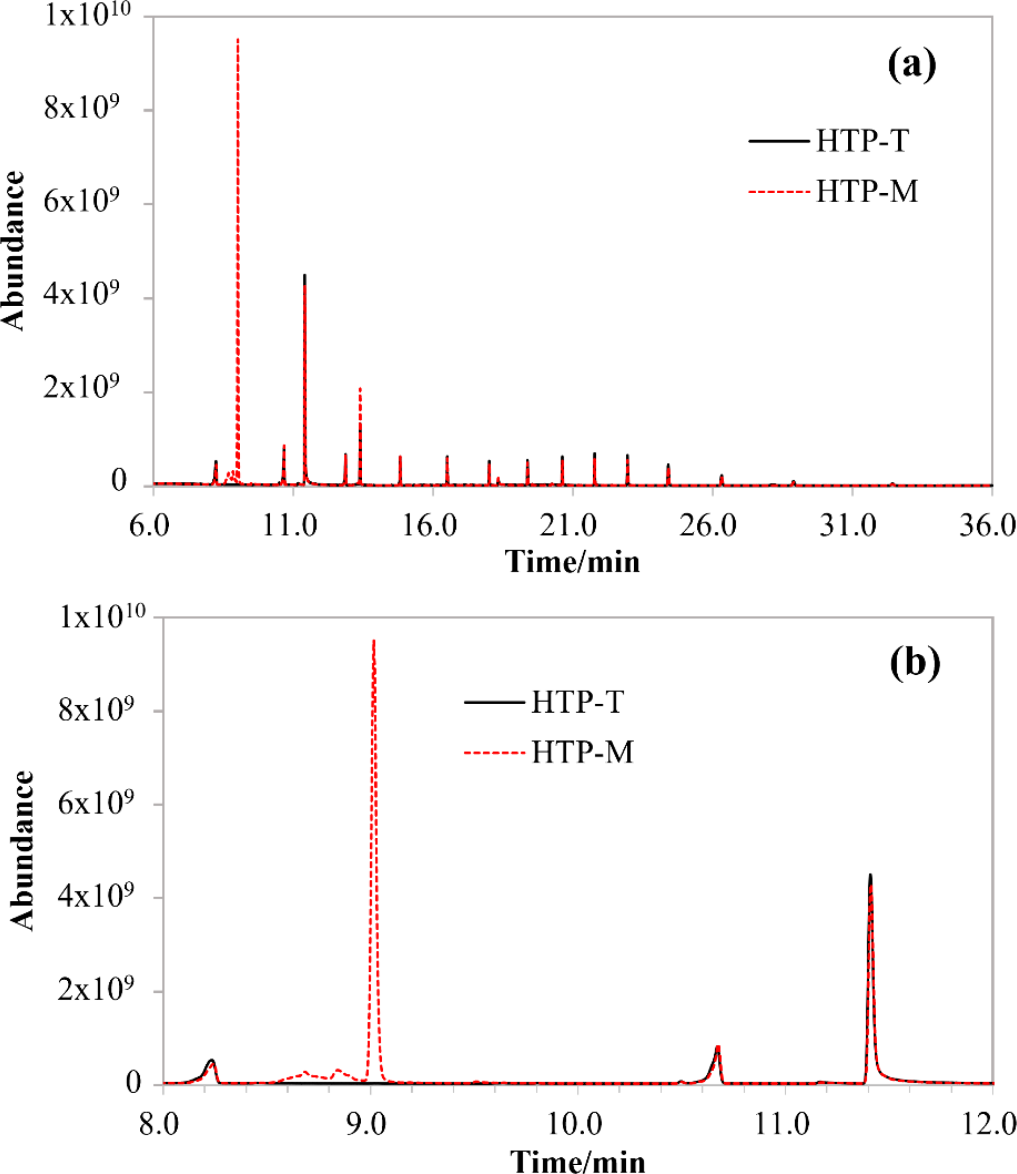
Comparison of GC-Orbitrap-MS Spectra of ACM Extracts: **(A)** Full Spectrum, **(B)** Zoomed-in Spectrum for Retention Times between 8–12 Minutes.

To explore the potential chemical components underlying the *in vitro* MAO inhibitory effects observed in HTP-T and HTP-M, a non-targeted chemical composition analysis was conducted using GC-Orbitrap-MS techniques (**Figure 3**). The overall spectral data indicated minimal differences between the aerosol collected mass (ACM) extracts of HTP-T and HTP-M (**Figure 3A**). However, computational analysis revealed that the most significant differences were associated with substances eluted between 8 and 12 minutes (**Figure 3B**). In total, 23 substances were detected in significantly higher quantities in HTP-M compared to HTP-T. Among these, the most pronounced difference was observed for menthol with a retention time of 9.015 minutes, which was detected exclusively in HTP-M but not in HTP-T. Additionally, two notable compounds were identified among the remaining 22 substances. The first, 3-Carboxy-N,3-diphenylpropionamide (eluted at 8.594 minutes, with a peak area ratio of HTP-M to HTP-T of 37, p<0.01), contains an amine group—a structural motif recognized by monoamine oxidase (MAO)—which may competitively interact with MAO substrates. The second, (2,5-dimethylpyrrolidin-1-yloxy)-N-phenylformamide (eluted at 8.907 minutes, with a peak area ratio of HTP-M to HTP-T of 5947, p<0.01), contains a N-phenylformamide functional group and could be a potential MAO inhibitor (30) warranting further investigation. These findings suggest that differences in MAO inhibitory potential between HTP-M and HTP-T may be attributed to the varying concentrations and structural characteristics of these chemical components.

### 3.3 Differential inhibitory effects of heated tobacco products on MAO-A activity

**Figure 4:**
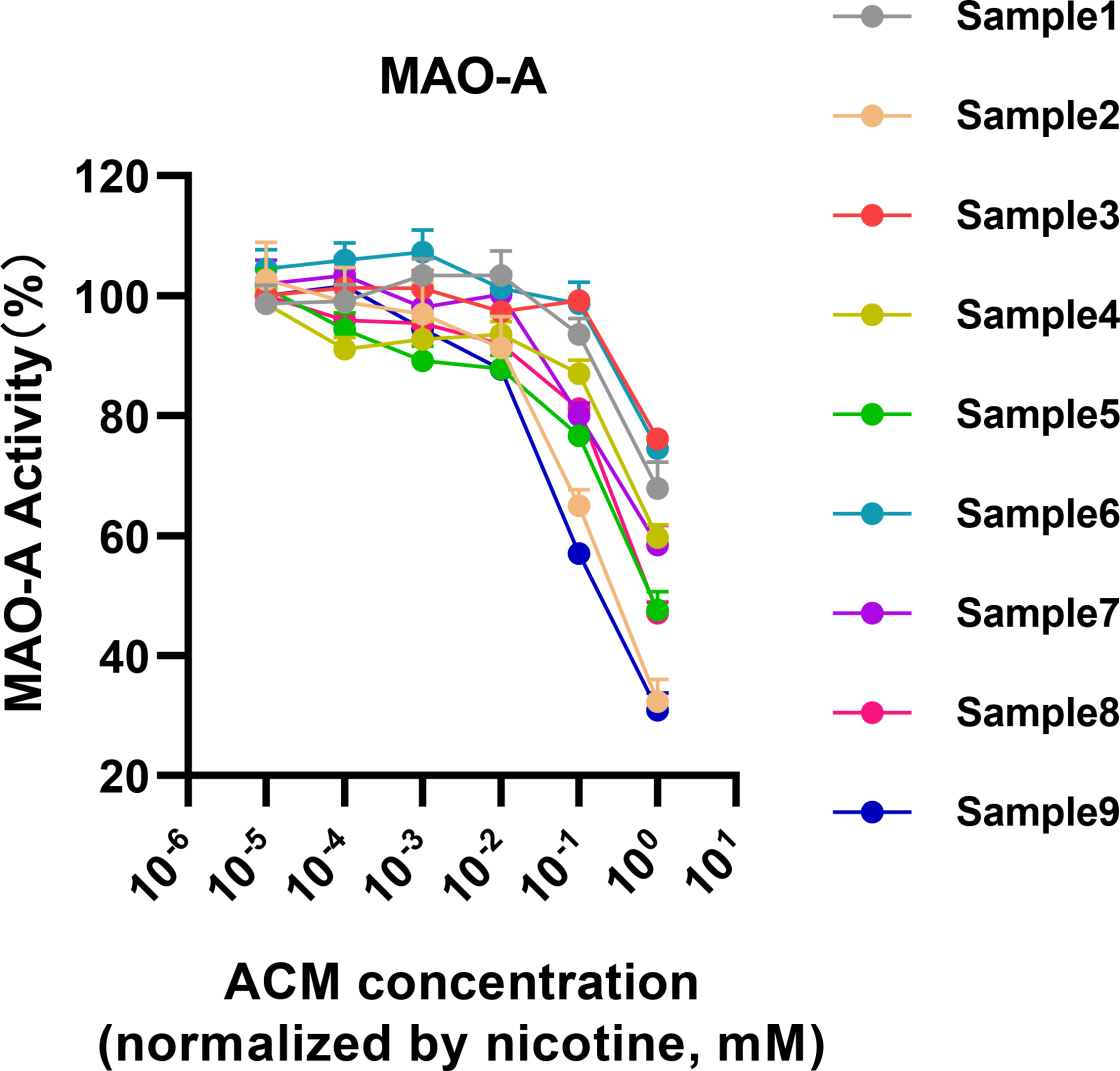
MAO-A inhibition by Heated Tobacco Products (HTPs) across different brands.

Expanding on the preliminary insights, we delved deeper into the specific inhibitory effects exerted by a diverse array of heated tobacco products (HTPs) on monoamine oxidase-A (MAO-A) activity. This analysis utilized the centrifugal method, which has been demonstrated as a superior approach for isolating active constituents from HTP emissions (as shown in **Figure 2**). The results of this comprehensive assessment indicated a relatively consistent inhibition of MAO-A across the spectrum of HTP brands tested, with nicotine concentrations ranging approximately from 10^-2^ to 10^-1^ mM serving as the inhibitory threshold (**Figure 4**). Notably, Sample 2 and Sample 9 demonstrated significantly stronger inhibitory effects on MAO-A compared to the other samples.

## 4 Discussion

A comprehensive evaluation of the HTP use is crucial, as HTPs become more widely publicized and the number of users increases. By employing a rat self-administration model to emulate human smoking behavior, this study offers a comparative analysis between HTPs—both tobacco and menthol-flavored—and nicotine in isolation. Our findings offer valuable insights into HTP use, particularly through the lens of monoamine oxidase (MAO) inhibition.

Our results indicate that HTP-T demonstrates a self-administration pattern comparable to that of nicotine under fixed-ratio (FR) schedules. These observations align with studies examining the subjective effects of HTPs, which report diminished craving alleviation and sensory impact (31). This may explain the poly-use phenomenon that users of HTPs may continue consuming other tobacco products (32). Notably, the self-administration results revealed a preference for HTP-M over HTP-T. Further *in vitro* analyses indicated that HTP-M exhibits slightly higher levels of MAO-A inhibition compared to HTP-T. Chemical analyses suggest that HTP-M aerosols contain more potential MAO inhibitors, warranting further investigation. The presence of MAO-A inhibitors in HTP-M aerosol may contribute to its higher self-administration levels relative to HTP-T. Additionally, menthol may enhance the reinforcing effects of nicotine by upregulating the expression and function of nicotinic acetylcholine receptors on dopamine neurons (33). This is further supported by evidence showing that menthol, at appropriate doses, can boost nicotine's reinforcement efficacy in self-administration tests (34)

A pivotal element of our research is the comparative analysis of MAO-A inhibitory effects between nicotine and HTPs. Our *in vitro* experiments indicate that both HTP-T and HTP-M, as used in the self-administration study, exhibited inhibitory effects at higher concentrations (>10^-2^~10^-1^ nicotine mM), with HTP-M showing slightly higher levels of MAO-A inhibition. In contrast, nicotine did not exhibit any MAO-A inhibition within the tested concentration range. These findings suggest that MAO-A inhibitors in HTP aerosol may still play a potential role in the rewarding effects of heated tobacco products.

Moreover, our examination of various HTP brands revealed considerable differences in MAO-A inhibition, suggesting that HTP brands vary significantly in the composition and quantity of MAO inhibitors. These differences may arise from factors such as heating temperatures, core material substrates, or the inclusion of non-tobacco ingredients. This underscores the importance of future research investigating brand-specific differences in HTPs. Interestingly, these findings diverge from previous studies, which reported no significant MAO-A inhibition in HTP aerosols compared to nicotine (15). This discrepancy may stem from the broader range of nicotine-equivalent concentrations tested in our study.

While this study advances our understanding of HTP use and brand-specific distinctions, certain limitations should be acknowledged. For instance, nicotine pharmacokinetic differences among HTP products were not examined, and the use of animal models and *in vitro* assays may not fully capture the real-world impact of MAO inhibition on HTP use. The idea of conducting dopamine tests during the process of nicotine self-administration in rats has been considered previously, but the simultaneous performance of surgeries on the brain and chest is extremely challenging. Even if survival is achieved, the required tubing is incompatible with the rats' natural movements, potentially increasing stress and discomfort for the animals. Future research should prioritize human studies to evaluate how MAO inhibition, along with the design and composition of various HTPs, influences nicotine pharmacokinetics and user behavior. Such investigations are vital to comprehensively understand the role of MAO inhibition in HTP use under real-world conditions.

## 5 Conclusions

This study offers valuable insights into heated tobacco products (HTPs), highlighting the role of monoamine oxidase (MAO) inhibition in their reinforcing effects. HTP-T exhibited self-administration patterns similar to nicotine, while HTP-M demonstrated stronger reinforcement, likely due to higher MAO-A inhibition and menthol's enhancement of nicotine's effects. Variability in MAO inhibition across HTP brands underscores the influence of non-nicotine constituents, highlighting the need for brand-specific research. While our findings advance understanding of HTP use, limitations include the reliance on animal models and the exclusion of nicotine pharmacokinetics. Future human-centered studies are essential to fully elucidate the role of MAO inhibition in HTP use under real-world conditions.

## Data Availability

The raw data supporting the conclusions of this article will be made available by the authors, without undue reservation.
