# Supplementary material for: Reinforcement and MAO-A inhibition in heated tobacco products: flavor and brand variations
Source: Front Psychiatry. 2025 Feb 18;16:1515519. doi: 10.3389/fpsyt.2025.1515519 (PMC11876406; doi:10.3389/fpsyt.2025.1515519)
Supplement: Supplementary file 1 [file DataSheet1.docx]

**Reinforcement and MAO-A Inhibition in Heated Tobacco Products: Flavor and Brand Variations**

LI Xiangyu^1,2^, DING Zheng^1,3^, JIANG Xingyi^1,2^, WANG Hongjuan^1,2^, LUO Yanbo^1,2^, CHEN Huan^1,2^, PANG Yongqiang^1,2^, HOU Hongwei*^1,2^, HU Qingyuan*^1,2^

1. China National Tobacco Quality Supervision &Test Center, Zhengzhou, Henan, China,450001

2. Key Laboratory of Tobacco Biological Effects, Zhengzhou, Henan, China,450001

3. Key Laboratory of Precision Nutrition and Food Quality, Department of Nutrition and Health, China Agricultural University, Beijing, China,100190

Figure S1 Comparision of Nicotine and Saline Control Self-Administraion in Rats across Progressive Fixed-Ratio (FR) Schedules (FR1 From Day 1 to Day 4, FR2 from Day 5 to Day 12, FR3 from Day 13 to Day 27)

Table S1: Content of ACM, Nicotine, and Menthol in the Mainstream Aerosol of Various Heated Tobacco Product (HTP) Brands

| Sample | ACM | nicotine | menthol |
| --- | --- | --- | --- |
| ID | mg/stk | mg/stk | mg/stk |
| sample 1 | 30.63 | 1.05 | N.D.* |
| sample 2 | 29.57 | 0.72 | N.D. |
| sample 3 | 25.93 | 2.10 | N.D. |
| sample 4 | 29.97 | 1.19 | N.D. |
| sample 5 | 30.40 | 0.81 | N.D. |
| sample 6 | 42.63 | 1.35 | N.D. |
| sample 7 | 44.93 | 2.38 | N.D. |
| sample 8 | 24.53 | 0.93 | N.D. |
| sample 9 | 27.20 | 1.23 | N.D. |

*N.D.=Not detected.
